# The Emerging Role of Epigenetics in Autoimmune Thyroid Diseases

**DOI:** 10.3389/fimmu.2017.00396

**Published:** 2017-04-07

**Authors:** Bin Wang, Xiaoqing Shao, Ronghua Song, Donghua Xu, Jin-an Zhang

**Affiliations:** ^1^Department of Endocrinology, Jinshan Hospital of Fudan University, Shanghai, China; ^2^Department of Rheumatology and Immunology, Jinshan Hospital of Fudan University, Shanghai, China; ^3^Department of Rheumatology and Immunology, The Affiliated Hospital of Weifang Medical University, Weifang, China

**Keywords:** epigenetics, autoimmune thyroid diseases, pathogenesis, DNA methylation, histone modifications, microRNAs, long non-coding RNAs

## Abstract

Autoimmune thyroid diseases (AITD) are a group of both B cell- and T cell-mediated organ-specific autoimmune diseases. Graves’ disease and Hashimoto thyroiditis are the two main clinical presentations of AITD. Both genetic and environmental factors have important roles in the development of AITD. Epigenetics have been considered to exert key roles in integrating those genetic and environmental factors, and epigenetic modifications caused by environmental factors may drive genetically susceptibility individuals to develop AITD. Recent studies on the epigenetics of AITD have provided some novel insights into the pathogenesis of AITD. The aim of this review is to provide an overview of recent advances in the epigenetic mechanisms of AITD, such as DNA methylation, histone modifications, and non-coding RNAs. This review highlights the key roles of epigenetics in the pathogenesis of AITD and potential clinical utility. However, the epigenetic roles in AITD are still not fully elucidated, and more researches are needed to provide further deeper insights into the roles of epigenetics in AITD and to uncover new therapeutic targets. Although there are many studies assessing the epigenetic modifications in AITD patients, the clinical utility of epigenetics in AITD remains poorly defined. More studies are needed to identify the underlying epigenetic modifications that can contribute to accurate diagnosis of AITD, adequate choice of treatment approach, and precise prediction of treatment outcomes.

## Introduction

Autoimmune thyroid diseases (AITD) are a group of both B cell- and T cell-mediated autoimmune diseases, which are caused by loss of immune tolerance and autoimmune attack to thyroid tissues (1, 2). The prevalence of AITD including Graves’ disease (GD) and Hashimoto’s thyroiditis (HT) is more than 5%, but the prevalence of thyroid autoantibodies is more than 10% in general population (1, 3). Despite its high prevalence, the incidence and prevalence of AITD have increased obviously in recent years (1, 4, 5). GD is the main cause of clinical hyperthyroidism, and HT is the main cause of clinical hypothyroidism (1, 6). Besides, AITD can also increase the risk of non-thyroid diseases, such as cardiovascular diseases, cancers, and adverse pregnancy outcomes (7–13). In addition, there is still lack of major breakthrough in the treatment of AITD (1, 2, 6, 14). Therefore, AITD have become a serious harm to public health, and more studies are urgently needed to explore the pathogenesis of AITD and develop new therapeutic strategies to effectively treat AITD.

Although the clinical manifestations of GD and HT are different, loss of immune tolerance caused by immune imbalance is believed to exert critical roles in the development of both diseases (15–17). Both GD and HT are characterized by the presence of autoantibodies against thyroid tissues, such as thyroid-stimulating hormone receptor antibody (TRAb), thyroid peroxidase antibody (TPOAb), and thyroglobulin antibody (TgAb) (15–17). TRAb is thyroid-stimulating hormone receptor (TSHR)-directed autoantibodies, and it can be classified as TSHR-stimulating autoantibodies, TSHR-blocking autoantibodies, or neutral depending on their respective abilities to induce cAMP generation and thyrocyte proliferation or bind to the receptor without impacting cAMP generation (18, 19). TPOAb and TgAb are the major autoantibodies in HT, but they also exist in some GD patients. The TRAb is the major autoantibody in GD patients, but it also usually occurs in some HT patients.

**Figure 1:**
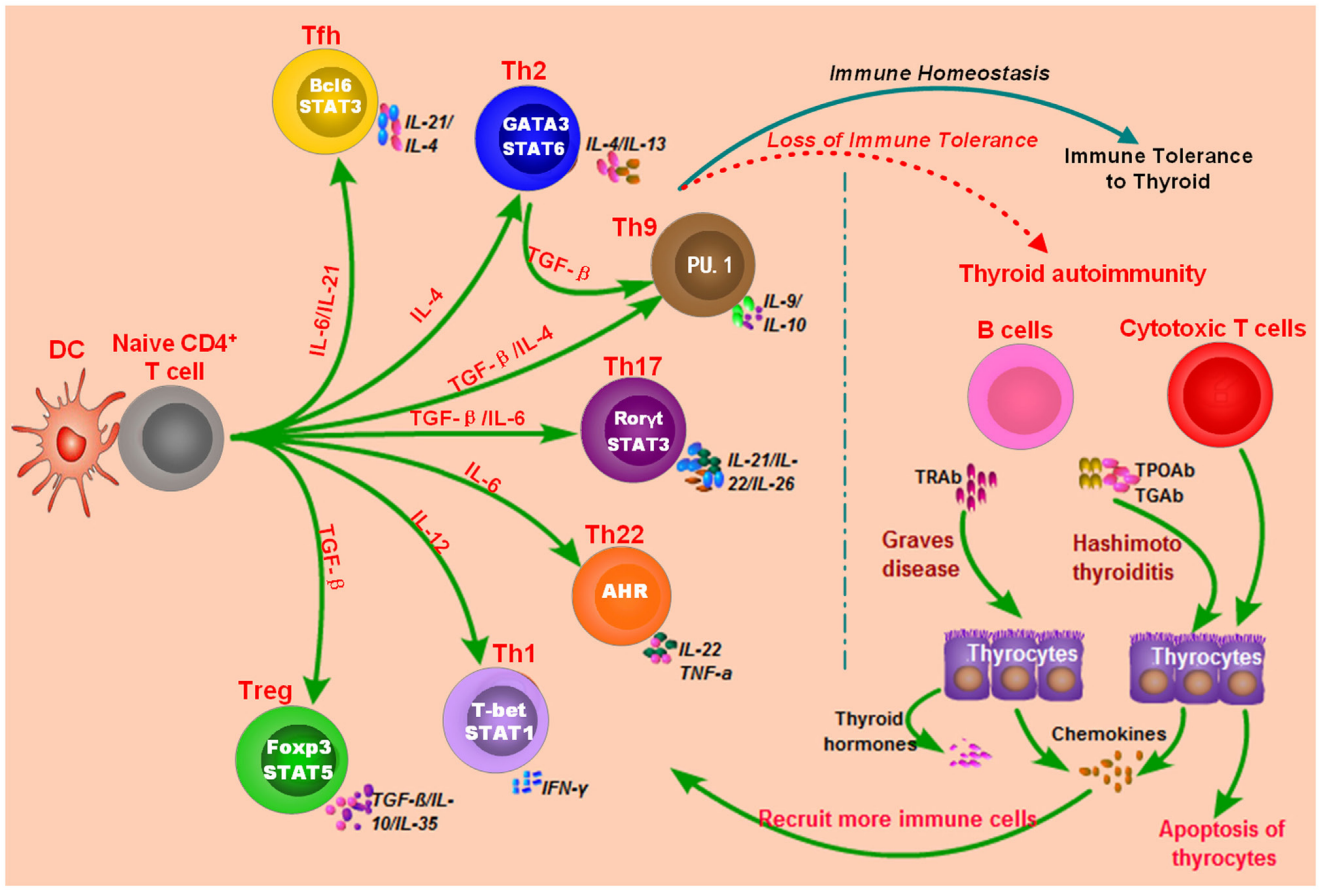
**Loss of immune tolerance results in autoimmunity during the development of autoimmune thyroid diseases**. Naive CD4+ T cells can be activated by dendritic cells (DC) or other antigen-presenting cells and they can differentiate into various subsets which are characterized by different cytokines and specific transcription factors. The balance of those immune cells is necessary for the maintenance of immune homeostasis. Under normal conditions, T cell subsets have normal functions, and there is immune homeostasis in human body, which can maintain the immune tolerance and avoid unwarranted immune attacks to thyroid tissues. Some genetic factors and environmental factors can result in the dysfunctions of these T cell subsets, B cells, and antigen-presenting cells, which may break up the immune homeostasis and cause thyroid autoimmunity.

Both GD and HT are characterized by lymphocytic infiltration in the thyroid tissues, and T cells and B cells can infiltrate into the thyroid gland during the development AITD (17, 20–22). Autoantibodies and B cell dysfunction are thought to be the primary immune reactions in AITD, and aberrant functions of T cell subsets also exert important roles in breaking the immune homeostasis and causing autoimmunity against thyroid tissues (17, 20–22) (Figure 1). Besides, T cells may amplify autoimmunity against thyroid by secreting pro-inflammatory cytokines, promoting B cells to generate more autoantibodies, and maintaining the number of autoreactive memory T cells against thyroid tissues (17, 20, 21, 23). In GD, the CD4+ T helper (Th) cells are important in the onset of the disease, and the roles of Th1 and Th2 in the development of AITD have also long been recognized in previous studies (24–27). Recent studies have suggested that the balance between T effector cells and T regulatory cells are important to maintain the immune tolerance to thyroid, and its imbalance can result in the development of AITD (22, 28, 29). Besides, the roles of other T subsets in AITD, such as Th17, Th22, and follicular helper T (Tfh) cells, have also been found in numerous researches (25, 30–34). Th17 cells are characterized by the transcription factors of signal transducer and activator of transcription 3 and Rorγt, and they mainly secret interleukin (IL)-17 (35). Increased circulating Th17 cells have been found in AITD patients, and IL-17 has also been identified as an important cytokine in the pathogenesis of AITD (25, 32–34, 36). Tfh cells are characterized by the expression of CXC chemokine receptor 5 (CXCR5) and the production of IL-21 and are critical for the activation of B cells and germinal center formation (37). Th22 cells are characterized by the transcription factor of aryl hydrocarbon receptor and the production of IL-22 (38). Increased proportions of circulating Th22 cells and Tfh cells and elevated of related cytokines in the AITD patients have been found in several published studies, suggesting the pathogenic roles of Th22 and Tfh in the pathogenesis of AITD (30, 31, 39). However, the molecular mechanisms underling the abnormal functions of those immune cells and immune imbalance during the development of autoimmune attacks to thyroid tissues are still unclear.

Autoimmune thyroid diseases are multifactorial and complex diseases, and multiple factors are involved in the development of AITD (1, 16). Studies in the past decade have proven the important role of genetic factors in the pathogenesis of AITD. In the past two decades, genetic studies in AITD have developed from candidate gene analyses, whole-genome linkage screening, genome-wide association study (GWAS), whole-genome sequencing, and epigenetic studies (16, 40–42). Some thyroid-specific genetic factors are found to be associated with AITD, such as polymorphisms in *TSHR* gene and *thyroglobulin (TG)* gene (43–45). Emerging evidence has suggested the important role of immunogenetics in the pathogenesis of AITD, and polymorphisms in these immune-modulating genes can impair immune tolerance and alter T cells’ interactions with antigen-presenting cells during the development of AITD (16, 46). Some immune-modulating genetic factors are also reported to be associated with AITD, such as polymorphisms in *HLA-DR3, CTLA4, PTPN22*, and *FOXP3* (47–49). Of those AITD susceptibility genes, *FOXP3* and *CD25* play critical roles in the establishment of peripheral tolerance, and *CD40, CTLA4*, and the *HLA* genes are pivotal for T lymphocyte activation and antigen presentation (16, 42). Those immune-modulating genetic factors can cause dysfunction of immune cells and loss of immune homeostasis, which can further result in the development of AITD. However, those genetic factors cannot fully explain host’s predisposition to AITD, and environmental factors also have important roles in AITD (16, 50, 51). The lack of full concordance in monozygotic twins also proves the importance of environmental factors in AITD (52, 53). Several environmental factors such as high iodine intake and vitamin D deficiency are proven to be risk factors of AITD (54–58). The genetic and environmental factors may cooperate together and cause the dysfunctions of immune cells and thyroid autoimmunity, but the mechanisms involving the effects of genetic and environmental factors on the immune cells’ function and immune balance are still not well understood (59–61).

Recent studies propose that environmental factors can interact with susceptibility genes to produce a synergistic effect in triggering diseases through epigenetic modulation (62–64). Epigenetics aim to study how non-genetic factors regulate the gene expressions and phenotypes and their roles in the development of diseases without involving alterations of the DNA sequence (65). Major epigenetic mechanisms mainly include DNA methylation, histone modifications, and RNA interference through non-coding RNAs (65). For example, DNA methylation can cause inactivation of genes, and some histone modifications can lead to activation of genes, but these factors are usually dynamic and can be affected by environmental factors (65–67). In addition, non-coding RNAs, such as microRNAs (miRNAs) and long non-coding RNAs (lncRNAs), can also regulate the expressions of targeted genes (68, 69). Therefore, genes involved in the immune system or thyroid can be regulated by epigenetic mechanisms, and dysfunctions of these genes caused by epigenetics can further result in autoimmune diseases. In the past decade, epigenetics have been considered to have key roles in integrating genetic and environmental factors in human complex diseases including autoimmune diseases (64, 70, 71). In the past decade, increasing evidence has suggested the critical roles of epigenetics in the pathogenesis of AITD, and epigenetic modifications caused by environmental factors may drive genetically susceptibility individuals to develop AITD (42, 60, 72–75). The aim of this review is to provide an overview of recent advances in the epigenetic mechanisms of AITD, to highlight the epigenetic roles in the pathogenesis of AITD, and to discuss the potential clinical utility of epigenetic modifications in AITD.

## Epigenetics in AITD

### DNA Methylation and AITD

DNA methylation is the most common type of DNA modifications, and it mainly occurs at the fifth carbon ring of cytosine in palindromic cytosine-phosphate-guanine dinucleotides (76–78). DNA methylation mainly results in transcriptional repression, especially when it occurs in the region of 5′ promoter regions with high density (79). In addition, methyl-binding domain family can recognize the methyl-CpG and result in transcriptional repression (79). Some important enzymes involved in DNA methylation have been found, such as DNA methyltransferases (DNMTs) and ten-eleven translocation (TET) enzymes for demethylation (78). In addition, DNA methylation can be reversed by TET enzymes, and the dynamic turnover of DNA methylation may be modulated through the relative expressions of DNMTs and TET enzymes (77, 78). Resent researches have provided evidence for the critical roles of DNA methylation in many autoimmune diseases through regulating gene expressions (80–85). Some agents targeting DNA methylation also provide promising novel treatment strategies for human diseases (86–88).

In the past decade, increasing evidence has demonstrated the roles of epigenetic dysregulation in the pathogenesis of AITD. Several studies have shown that global DNA hypomethylation exists in AITD patients, which may cause the overexpression of some genes involved in immune function or the activation of immune cells and further result in autoimmune attack toward thyroid tissues (74, 75). We previously studied the genome-wide DNA methylation of GD patients and revealed more than 200 hypermethylated and hypomethylated genetic regions in GD patients, such as *ICAM1, DNMT1*, and *MECP2* genes (74). Limbach et al. investigated the genome-wide DNA methylation of CD4+ and CD8+ T cells of GD patients and found more than 300 differentially methylated sites in CD4+ T cells and more than 3,000 differentially methylated sites in CD8+ T cells, and many of those genes were from T cell signaling (75). Many of those DNA methylations were immune-related modifications, such as hypermethylated sites in *ICAM1, CD247*, and *CTLA4* (74, 75). Limbach et al. also found hypermethylation of the first intron area in *TSHR* gene was associated with GD, which suggested methylation in thyroid-specific genes could also be involved in the development of AITD. The above findings demonstrated the existence of aberrant DNA methylation at some genes during the development of AITD. However, the molecular mechanisms underlining the roles of those differentially methylated genes are still unclear.

Some genetic polymorphisms of DNA methylation-regulating genes can also cause dysfunction of these genes and aberrant DNA methylation, which further increases hosts’ susceptibility to diseases (89, 90). Arakawa et al. reported that several polymorphisms in methylation-regulating genes, such as *DNMT1* and methionine synthase reductase (*MTRR*), were correlated with DNA hypomethylation levels and susceptibility to AITD (91). Our previous study also found evidence for a potential role of *DNMT3B* rs2424913 and *DNMT1* rs2228611 in the susceptibility to AITD (92). 5,10-Methylenetetrahydrofolate reductase C677T polymorphism was also associated with GD and Graves’ ophthalmopathy (93, 94). The above findings also provide secondhand evidence for the important roles of DNA methylation in the pathogenesis of AITD.

The above findings suggest the emerging and important roles of DNA methylation in AITD, but current understanding on DNA methylation in AITD is still very limited. More studies in the future are necessary to further explore the possible key roles of DNA methylation in AITD and new promising treatment strategies targeting DNA methylation for AITD patients. In addition, the clinical utility of DNA methylation in AITD as biomarkers for disease diagnosis and predictors of treatment outcomes is still unclear, which need to be explored in future studies.

### Histone Modifications and AITD

Histone has key roles in the compaction of DNA, interacting with DNA to form tightly packed chromatin, and it also has been suggested to have critical roles in many human diseases (95–98). The unstructured *N*-terminal amino acids in histones can be modified and then affect the chromatin structure and function directly or by binding some protein effectors (99). These modifications usually occur at the lysine or arginine of histones and are mediated by histone-modifying enzymes, which are intensively involved with chromosome remodeling (98). There are several main types of modifications in histones, such as methylation, acetylation, deacetylation, phosphorylation, ubiquitination, and sumoylation (99, 100). Histone modifications have important roles in controlling chromatin compaction, nucleosome dynamics, and DNA repair, and it can directly regulate transcription (101, 102). Like DNA methylation, histone modifications are highly dynamic and are regulated by “writer” and “eraser” enzymes (77, 100, 103). Recent researches have provided some evidence for the roles of histone modifications in modulating immune tolerance and autoimmune diseases (104–109). Some small-molecule inhibitors targeting histone-modifying enzymes, such as histone deacetylases (HDAC), also provide new treatment strategies for diseases such as cancers and autoimmune diseases (87, 110).

We found that the histone H4 acetylation level in the peripheral blood mononuclear cells of GD patients was significantly lower than that of healthy individuals, but the levels of HDAC1 and HDAC2 were obviously higher, which proved evidence for the aberrant histone modifications in GD patients (104). A genome-wide analysis by Limbach et al. found decreased levels of H3K4me3 and H3K27ac at several genes of T cell signaling in GD patients (75). In addition, phosphorylated histone protein H2A.X was also observed in the T cells and thyrocytes in the mice of AITD model (111).

Stefan et al. found that interferon-alpha (IFN)-α could induce alterations of *TG* gene expression and trigger AITD through enrichment of Lys-4 residue methylation in histone H3 at the promoter area of *TG* gene (112). IFN-α is a key cytokine secreted during viral infections, and it has been found to increase levels of H3K4me3 and H3K4me1 in thyroid cells (72). Another study by Kawashima et al. showed that DNA fragments released during thyroid injury could be recognized by histone H2B, which further resulted in the activation of genes of immune responses and triggered autoimmunity against thyroid tissue (113).

Genetic polymorphisms of histone-modifying genes can also cause dysfunction of these genes and aberrant histone modifications (114–116), which may further results in AITD. Sirtuin1 (SIRT1) is a class 3 nicotinamide adenine dinucleotide-dependent HDAC, which is intensively associated with immune response and autoimmune diseases (114–116). A case–control study by Sarumaru et al. reported that rs3758391 and rs4746720 in the SIRT1 gene were associated with higher levels of thyroid autoantibodies in AITD patients (117).

The findings above suggest the important role of histone modifications in AITD, but they are still not fully elucidated. More researches are needed to further elucidate the roles of histone modifications in AITD. Besides, the roles of histone modifications as diagnostic biomarker and predictors of treatment outcomes in AITD patients have also not been investigated, and future researches are recommended to study these roles of histone modifications in AITD.

### miRNAs and AITD

microRNAs are endogenous small non-coding RNAs ranging from 18 to 25 nucleotides in length and have important roles in regulating gene expression (118). miRNAs can regulate about 60% of all mRNAs and are involved in many diseases, such as cancers, metabolic diseases, and inflammatory diseases (118–120). Some miRNAs also play important roles in regulating immune function and maintaining immune homeostasis, such as miR-223-3p and miR-155-5p (120–122). It is not surprising that abnormal expressions of miRNAs involving immune function can potentially contribute to the development of autoimmune diseases (123, 124). Recent studies have revealed that some miRNAs are also involved in the development of AITD, and most of them have been found to be intensively involved in modulating the differentiation or activation of immune cells and immune response (Table 1).

**Table 1:** **Aberrant expressions of non-coding RNAs in AITD patients**.

Diseases	Samples or cells	Expression changes	Epigenetic alteration/function	Reference
GD	Serum	Increased expression of miR-346 in GD patients with relapse	Predicative factor for relapse	(125)
AITD	Circulating microvesicles	Upregulation of miR-146a-5p and miR-155-5p	Possibly targeting IL-8 and SMAD4	(126)
GD	PBMC	Downregulation of lncRNAs Heg	Disease biomarker and possibly decreased CD14 mRNA level of mononuclear cells	(127)
GD	Thyroid tissues	Downregulation of miR-146a-5p	Unclear	(128)
GD	PBMC	Downregulation of miR-154-3p, miR-376b-3p, and miR-431-3p	Disease biomarker	(129)
GD	CD4+ T cells and CD8+ T cells	Downregulation of miR-200a-3p and miR-200a-5p in both CD4+ T cells and CD8+ T cells; Downregulation of miR-155-5p and miR-155-3p in CD8+ T cells	Disease biomarker	(130)
GD	PBMC	Downregulation of miR-125a-5p	Disease biomarker	(131)
GD	Regulatory T cells	Upregulation of miR-155-5p, miR-519e-5p, and miR-30a–5p; Downregulation of miR-19b-3p and miR-146a-5p	Disease biomarker; possibly regulating retinoic acid pathway	(60)
GD	Serum	Upregulation of miR-451a, miR-16-5p, miR-22-3p, and miR-375	Disease biomarker	(73)
GD	Serum, CD4+ T cells	Downregulation of miR-346	Disease biomarker; regulating CD4+CXCR5+ T cells by targeting Bcl-6	(132)
GD	Thyroid tissues	5 unregulated miRNAs, such as miR-22-3p and miR-183-5p, and 18 downregulated miRNAs, such as miR-101-3p, miR-660-5p, and miR-197-3p	Possible miRNA-target gene network	(133)
GD	Serum	MiR-23b-5p and miR-92a-3p were significantly increased in GD patients achieving remission, while let-7g-3p and miR-339-5p were significantly decreased in GD patients achieving remission.	Biomarkers of clinical activity	(134)
Graves’ ophthalmopathy	Serum	Lower serum level of miR-224-5p was independently associated with glucocorticoid insensitivity	Biomarker of glucocorticoid insensitivity	(135)
Graves’ ophthalmopathy	Orbital fibroblasts	Upregulation of miR-21-5p	Activating the TGF-beta1/Smad signaling pathway by enhancing Smad3 phosphorylation	(136)
Graves’ ophthalmopathy	Serum	Downregulation of miR-146a-5p	Disease biomarker; being correlated with the clinical activity	(137)
Graves’ ophthalmopathy	PBMC	Downregulation of miR-146a-5p and upregulation of miR-155-5p	Disease biomarker	(138)
HT	PBMC	Upregulation of lncRNA IFNG-AS1	Disease biomarker; contributing to Th1 cell response possibly through regulating the expression of IFN-γ	(139)
HT	PBMC	Upregulation of let-7e-5p	Disease biomarker; possibly regulating IL-10 expression	(140)
HT	Thyroid tissues; serum	Upregulation of miR-142-5p, miR-142-3p, and miR-146a-5p in thyroid tissues; upregulation of miR-142-5p in the serum	MiR-142-5p regulated the expression of claudin-1 and increased permeability of thyrocytes	(141)
HT	PBMC	Downregulation of miR-125a-3p	Disease biomarker; directly inhibiting interleukin-23 receptor expression	(142)
HT	Serum	Upregulation of miR-451a, miR-22-3p, and miR-375	Disease biomarker	(73)
HT	CD4+ T cells and CD8+ T cells	Downregulation of miR-200a-3p and miR-200a-5p in both CD4+ T cells and CD8+ T cells; downregulation of miR-155-5p and miR-155-3p in CD8+ T cells	Disease biomarker	(130)
HT	Thyroid tissues	Downregulation of miR-155-5p and upregulation of miR-200a-3p	Unclear	(128)
HT	Thyroid tissues	Downregulation of miR-141-3p	Possibly regulating the TGF-beta pathway	(143)

*AITD, autoimmune thyroid diseases; GD, Graves’ disease; HT, Hashimoto’s thyroiditis; IL, interleukin; IFN, interferon; miRNAs, microRNAs; lncRNA, long non-coding RNA; PBMC, peripheral blood mononuclear cells; TGF-beta, transforming growth factor-beta*.

**Figure 2:**
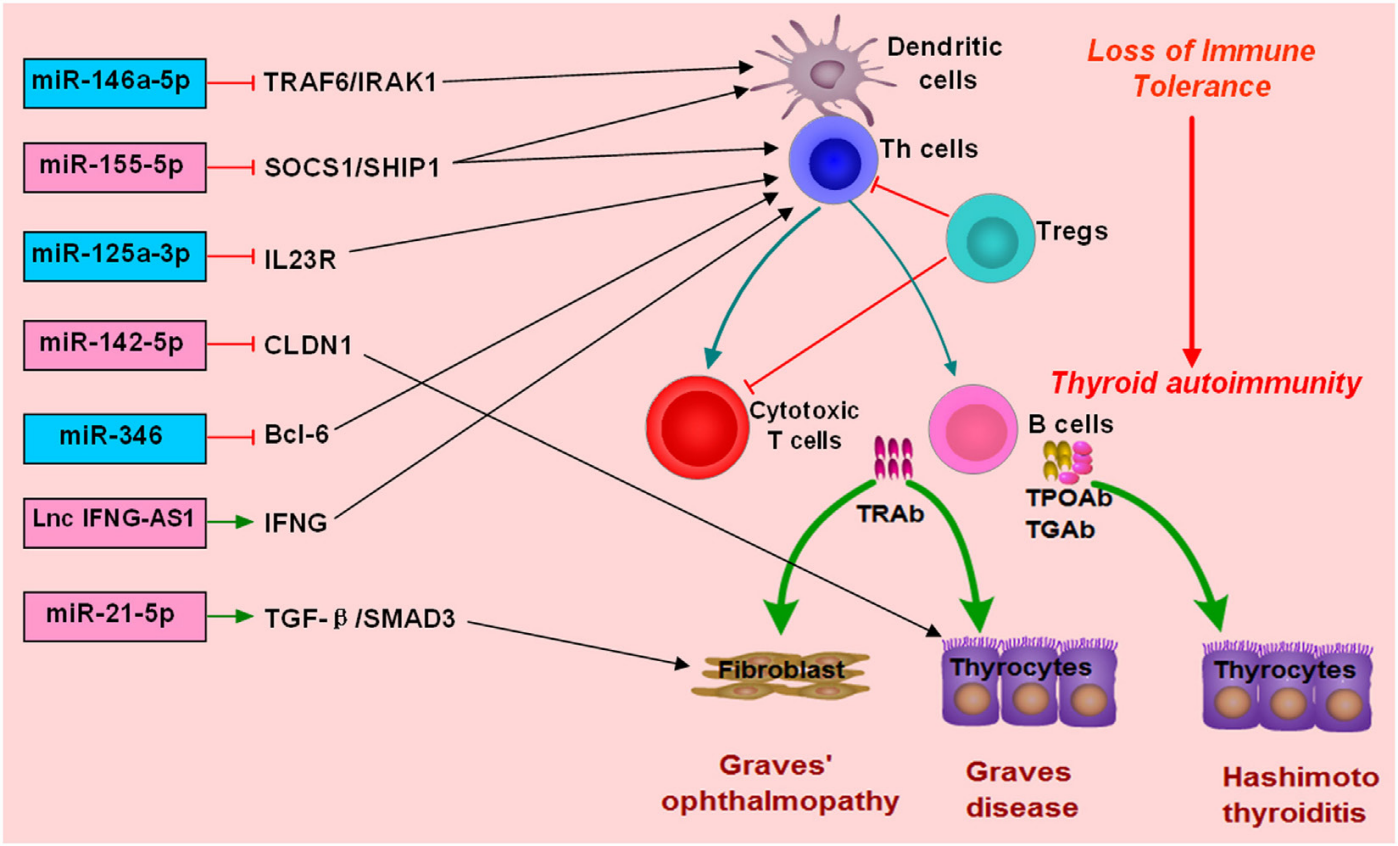
**Roles of non-coding RNAs in the development of autoimmune thyroid diseases (AITD)**. microRNAs (miRNAs) or long non-coding RNAs (lncRNAs) can target some genes involved in immune response or the function of immune cells. The altered expression of miRNAs or lncRNAs can alter the normal function of immune cells, break immune homeostasis, and result in immune attacks toward thyroid tissues during the development of AITD. MiR-146a-5p can repress IL-1R-associated kinase 1 (IRAK1) and TNF-receptor-associated factor 6 (TRAF6), and its downexpression will increase the activation and antigen presentation of dendritic cells. Other miRNAs, such as miR-125-3p and miR-346 and miR-155-5p, can also regulate the immune functions of Th cells or dendritic cells by targeting transcription factors or key molecules. Lnc IFNG-AS1 can increase the expression of IFNG and increase the activation Th1 cell, and increased level of lncRNA IFNG-AS1 thus contributes to Th1 cell response in HT patients. MiR-21-5p can promote collagen I expression and total collagen production induced by TGF-beta1 in orbital fibroblasts, and increased expression of miR-21-5p thus can contribute to Graves’ ophthalmopathy. MiR-142-5p can target CLDN1, and its overexpression in thyrocytes can result in reduced expression of claudin-1 and increased permeability of thyrocytes monolayer.

MiR-155-5p and miR-146a-5p are two widely studied miRNAs and have important roles in modulating immune response (122, 144–146). MiR-146a-5p could repress IL-1R-associated kinase 1 and TNF-receptor-associated factor 6, and its downexpression would increase the activation and antigen presentation of dendritic cells (144, 147). MiR-155-5p also could regulate the immune functions of Th cells or dendritic cells by targeting transcription factors or key molecules involved in immune response (122, 148, 149). Abnormal expressions of miR-155-5p and miRNA-146a-5p can contribute to the development of autoimmune diseases by breaking immune homeostasis and immune tolerance. There are several studies providing evidence for the abnormal expression of miR-155-5p and miR-146a-5p in AITD patients (Figure 2; Table 1). Bernecker et al. found that miR-146a-5p and miR-155-5p were differently expressed in the thyroid tissues of AITD (128). GD and HT patients had significantly lower levels of miR-146a-5p and miR-155-5p in the thyroid tissues, respectively (128). A subsequent study by Bernecker et al. found that GD and HT patients had lower level of miR-155-5p in HT in CD8+ T cells than controls (130). Wei et al. reported that Graves’ ophthalmopathy patients had significantly lower levels of miR-146a-5p than controls, and miR-146a-5p was negatively correlated with serum level of IL-17, which had been suggested to be an important pathogenic cytokine in the development of Graves’ ophthalmopathy (33, 34, 137). However, few studies have explored their clinical utility as diagnostic biomarkers or predictors of treatment outcomes. Besides no study has been performed to explore the feasibility of treatment strategies targeting those two miRNAs in AITD, especially for those with intractable GD.

There are also some other miRNAs found to be associated with AITD (Table 1). Chen et al. found downregulation of miR-346 in GD patients, and miR-346 could inhibit Bcl-6 expression and regulate the activation of CD4+CXCR5+ T cells (132). Zhu et al. reported that miR-142-5p was highly expressed in HT patients and was positively correlated with TgAb (141). Overexpression of miR-142-5p in thyrocytes resulted in reduced expression of claudin-1 and increased permeability of thyrocytes monolayer (141). Tong et al. found that the expression of miR-21-5p in orbital fibroblasts from Graves’ ophthalmopathy was higher than that in the controls, and miR-21-5p could promote collagen I expression and total collagen production induced by TGF-beta1 in orbital fibroblasts (136). Peng et al. found that miR-125a-3p could target IL-23 receptor (IL-23R), and its decreased expression of miR-125a-3p could upregulate IL-23R expression in HT patients (142). Our previous study simultaneously detected the expression profiles of miRNAs and mRNAs in the thyroid tissues of GD patients (133). We found five unregulated miRNAs, such as miR-22-3p and miR-183-5p, and 18 downregulated miRNAs in the thyroid tissues of GD patients, such as miR-101-3p, miR-660-5p, and miR-197-3p (133). The finding from our study highlighted a miRNA-target gene network in the pathogenesis of GD (133). Other abnormally expressed miRNAs identified in AITD patients could be found in Table 1.

Some studies also studied the clinical utility of miRNAs in AITD. Hiratsuka et al. used miRNA array to identify circulating miRNAs in relation to disease activity of GD by recruiting seven intractable GD patients, seven GD patients in remission, and seven healthy controls, and found that miR-23b-5p and miR-92a-3p were significantly increased in GD patients achieving remission, but let-7g-3p and miR-339-5p were significantly lower in GD patients achieving remission than intractable GD patients, demonstrating that some miRNAs could be as biomarkers of intractable GD and treatment response (134). A recent study by Li et al. found that patients with higher miR-346 level at diagnosis were at a higher risk of relapse during follow-up (125). A study by Shen et al. found that lower serum level of miR-224-5p was independently associated with glucocorticoid insensitivity in Graves’ ophthalmopathy, and *in vitro* overexpression of miR-224-5p could restore glucocorticoid sensitivity *via* targeting GSK-3beta (135). However, apart from those three studies above, no other studies assessing the clinical utility of miRNAs in AITD are available.

microRNAs are important regulators of gene expression, while genetic variants in miRNAs have been associated with many diseases (150–152). Inoue et al. found that MIR125A rs12976445 C/T was significantly associated with HD and intractable GD (131). Our recent study also proved that rs3746444 of miR-499a and rs12976445 of miR-125a-5p were associated with AITD susceptibility (153). The above study also indicated the roles of miRNAs in the pathogenesis of AITD. In addition, Dicer is an important ribonuclease involved in the biogenesis of miRNAs (154). Frezzetti et al. reported that the development of the thyroid gland was not affected by the absence of Dicer through using thyrocyte-specific Dicer knockout mice, but Dicer knockout resulted in severe hypothyroidism (155). In addition, Dicer inactivation also increased the expressions of Tg and decreased the expressions of cell adhesion proteins in the thyroid cells, such as Cdh16 and Cdh1 (155). Saeki et al. found that AITD patients had lower expression of Dicer and DROSHA compared with healthy controls, and DICER rs1057035 and DROSHA rs644236 were obviously associated with susceptibility to GD (156).

Although there are many studies investigating the differently expressed miRNAs in AITD patients, few studies have explored their clinical utility. The diagnostic values of those miRNAs and their roles in the risk stratification of AITD patients have not been clearly defined. More studies are needed to investigate the clinical significance of miRNAs and assess whether miRNAs can help to promote advances in the personalized therapeutics for AITD patients. Besides, no study has been performed to explore the feasibility of treatment strategies targeting miRNAs in AITD, especially for the treatment of intractable GD. Therefore, more studies in the future are needed to find more AITD-related miRNAs, to explore their molecular roles in the pathogenesis of AITD, and to investigate their clinical utility in AITD.

### LncRNAs and AITD

Long non-coding RNAs are a class of non-coding RNAs with a length of more than 200 nt, which are also involved in autoimmune diseases (157, 158). LncRNAs are found to be involved in a variety of biological processes, and lncRNAs may regulate gene expressions at various levels, such as epigenetic regulation, transcriptional regulation, posttranscriptional regulation, and regulating miRNAs (159–161). Many studies have confirmed that lncRNAs play critical roles in immune system development and function regulation (162, 163). LncRNAs are involved in regulating T cell production and differentiation, and different types of T cells may express certain specific lncRNAs (162, 164–166). Previous studies have reported aberrant expressions of many lncRNAs in various autoimmune diseases, but the precise mechanisms underling the roles of lncRNAs in autoimmune diseases are still largely unknown (167–170). There are also several studies suggesting the possible important roles of lncRNAs in AITD (127, 139, 171, 172).

Christensen et al. first reported one lncRNA associated with GD, namely lncRNA Heg (127). LncRNA Heg was correlated with the levels of TRAb and CD14 mRNA in the mononuclear cells of GD patients (127). *In vitro* studies suggested that lncRNA Heg could significantly decrease the level of CD14 mRNA of mononuclear cells (127). However, antithyroid treatment was unable to change the level of lncRNA Heg in GD patients (171). Peng et al. found that lncRNA-IFNG-AS1 was upregulated in HT patients, and it was associated with the frequency of circulating Th1 cells and IFN-γ expression (139). In addition, lncRNA-IFNG-AS1 could regulate IFN-γ expression in human CD4+ T cells and may promote Th1 response in the development of HT (139). A GWAS by Zhao et al. found a susceptibility locus of GD at an intergenic region harboring two non-coding RNAs at 14q32.2, and two lncRNAs including C14orf64 and GDCG14q32.2 were reported to be potentially involved in the pathogenesis of GD (172).

Researches on the lncRNAs associated with AITD will also provide potential drug targets and help us to find some novel treatment strategies for AITD (42, 173). Currently, there is still lack of a good understanding on the regulatory network of lncRNAs/miRNAs/mRNAs in the molecular mechanisms of AITD. More studies in the future are needed to find more AITD-related lncRNAs, to explore their roles in AITD in details, and to investigate the feasibility of non-coding RNA-based therapeutic agents for AITD (174). In addition, the clinical utility of lncRNAs in AITD patients is also poorly studied, and more studies are needed to identify those lncRNAs associated with different types of AITD and to assess their roles in predicting treatment responsiveness, guiding the choice of treatment approach for GD patients, and predicting relapse risk during follow-up.

### X Chromosome Inactivation (XCI) in AITD

X chromosomes are randomly inactivated in females, which can result in a mosaic pattern of cells expressing genes from either chromosome (175). XCI is a major epigenetic feature in which one X chromosome is transcriptionally silenced, and the X-inactive-specific transcript has major roles in the silencing (176, 177). Currently, histone modifications, DNA methylation, and non-coding RNAs are all considered to be involved in the formation of XCI (178). Skewed XCI occurs when the inactivation of one X chromosome is silenced more than the other one (178, 179). Skewed XCI can also result in loss of imbalance of gene products and immune tolerance and thus is involved in many autoimmune diseases (180–183).

Autoimmune thyroid diseases occur more often in females, suggesting a key role for the XCI in AITD (176). Skewed XCI has been proposed as a potential mechanism explaining the female preponderance of AITD (184). Previous studies have proven the increased frequency of skewed XCI in AITD patients (182, 184–187). Brix et al. first conducted a case–control study and found that the frequencies of skewed XCI in female twins with GD and HT were both significantly higher than the controls (11%) (185). Ozcelik et al. reported that extreme skewing of XCI was present in 19% of AITD patients, but only in 2.4% of controls (*P* < 0.0001) (182). Yin et al. also found that XCI skewing was significantly associated with AITD (OR = 4.0, *P* = 0.004) (186). However, a recent study by Ishido at al. reported that there was no obvious difference of skewed XCI between AITD cases and controls, but it was significantly higher in intractable GD patients (66.7%) than those with GD remission (25.0%), which suggested that skewed XCI was related to the GD progression (188).

## Summary

The important roles of epigenetics in AITD have been increasingly recognized, but many of them are still largely unknown, which need to be elucidated in more epigenetic researches in the future. Specially, more studies are needed to find more AITD-related epigenetic modifications, to explore deeper complex interactions of epigenetic factors in the pathogenesis of AITD, and to investigate the feasibility of epigenetic-based therapeutic strategies for the treatment of AITD. In addition, it is still unclear whether some thyroid-specific lncRNAs and miRNAs could have roles in the pathogenesis of AITD, and future studies are recommended to explore it. Currently, there are still great challenges in providing effective healthcare for AITD patients, such as adequate choice of treatment protocols for GD, precise prediction of treatment response, and appropriate personalized therapeutics for AITD. Epigenetics undoubtedly provide opportunities of a better understanding of the mechanism of AITD and may help to solve these challenges and promote advances in the personalized therapeutics of AITD. However, although there are many studies assessing the epigenetic modifications in AITD patients, few studies have explored those epigenetic modifications that are associated with types of AITD, treatment outcomes, and risk of relapse during follow-up, and the clinical utility of epigenetics in AITD remains poorly defined. A better understanding of those epigenetic modifications can contribute to accurate diagnosis of AITD, adequate choice of treatment approach, and precise prediction of treatment outcomes, and it’s recommended in future researches. Besides most studies available now focus on one type of epigenetic modifications (e.g., DNA methylation) in isolation, which is unlikely to fully explain the etiology of AITD. Hence, to get a better understanding of the etiology of AITD and improve the clinical utility of epigenetics in AITD, it is essential to integrate analyses of multiple epigenetic modifications together in future studies. The increasing use of bioinformatics and high-throughput sequencing will provide much help in interpreting analyses of multiple epigenetic modifications in AITD. Finally, clinical observational studies with large number of AITD patients are also needed, which will provide essential evidence for the clinical utility of those epigenetic modifications in AITD.

## Author Contributions

BW and JZ designed the study. BW, XS, RS, DX, and JZ contributed to the literature search, interpretation, writing, and proofreading of the manuscript. BW generated the figures.

## Conflict of Interest Statement

The authors declare that the research was conducted in the absence of any commercial or financial relationships that could be construed as a potential conflict of interest.
